# A Wideband GRIN Dielectric Lens Antenna for 5G Applications

**DOI:** 10.3390/mi14050997

**Published:** 2023-05-03

**Authors:** Khaled Aljaloud, Yosef T. Aladadi, Majeed A. S. Alkanhal, Wazie M. Abdulkawi, Rifaqat Hussain

**Affiliations:** Department of Electrical Engineering, King Saud University, Riyadh 11421, Saudi Arabia

**Keywords:** GRIN, dielectric flat lens, microstrip patch antenna, 5G

## Abstract

This paper proposes a graded effective refractive indexes (GRIN) dielectric lens for 5G applications. The inhomogeneous holes in the dielectric plate are perforated to provide GRIN in the proposed lens. The constructed lens employs a collection of slabs that correspond to the specified graded effective refractive index. The thickness and the whole lens dimensions are optimized based on designing a compact lens with optimum lens antenna performance (impedance matching bandwidth, gain, 3 dB beamwidth, and sidelobe level). A wideband (WB) microstrip patch antenna is designed to be operated over the entire band of interest from 26 GHz to 30.5 GHz. For the 5G mm-wave band of operation, the behavior of the proposed lens along with a microstrip patch antenna is analyzed at 28 GHz for various performance parameters, including impedance matching bandwidth, 3 dB beamwidth, maximum gain, and sidelobe level. It has been observed that the antenna exhibits good performance over the entire band of interest in terms of gain, 3 dB beamwidth, and sidelobe level. The numerical simulation results are validated using two different simulation solvers. The proposed unique and innovative configuration is well-suited for 5G high gain antenna solutions with a low-cost and lightweight antenna structure.

## 1. Introduction

The need for high data rates led to the use of millimeter-wave (MM-wave) bands in fifth-generation (5G) mobile systems [1]. The development of antenna lenses that operate at 5G frequencies is an important research topic aimed at improving the efficiency of 5G technology [2]. However, the 5G communication system faces many problems due to its large channel capacity, unique channel characteristics, hardware limits, concrete path, and penetration losses at millimeter wavelengths [3]. Due to these challenges, lenses offer an effective solution for establishing and maintaining a reliable millimeter-wave communication link.

For spherical lenses, the beam moving along the main axis is focused on a focal point [4]. So, when EM radiation passes through the lens, it can be transformed into a beam emanating from the source at the focal point. Isotropic spherical dielectric lenses have the benefit of focusing the light in both azimuthal and elevation planes. However, these lenses are bulky, heavy, challenging to fabricate, and require specialized mechanical or printing devices. The use of Fresnel zoned plate lenses reduces the thickness of isotropic spherical dielectric lenses; however, they suffer from low efficiencies and zone edge blockage [5]. Luneburg and Maxwell fisheye lenses are the most commonly used lenses at millimeter-wave frequencies [6]. Luneburg lenses possess a refractive index gradient that varies continuously, while Maxwell fisheye lenses have a refractive index profile that changes stepwise. These types of lenses have extensive applications in wireless communication, radar, and sensing. However, achieving the desired performance requires accurate control of the refractive index profile, making the design and fabrication of these lenses challenging. The effective refractive index varies in the range of 1–√2 for classical Luneburg lenses and in the range of 1–2 for Maxwell fisheye lenses. The transformation optics theory is typically used to develop a similar 2D lens based on the graded effective refractive indices [7]. Different focusing characteristics can be achieved by grading the refractive index of the lens’s material. The possible half-power beam width is inversely proportional to the spherical lens’s diameter (HPBW). A focusing beam in a constrained azimuthal or elevation plane has also been used to show the theoretical conversion of 3D lens action on the 2D surface [8]. For forming lenses in the microwave and sub-terahertz regimes, metallic parallel-plate waveguide structures are considered the simplest periodic artificial sub-wavelength structures with a graded effective refractive index (GRIN) [9]. However, metallic lenses show anisotropic behavior, have a narrow bandwidth and are costly and complex [10]. GRIN materials are materials in which the refractive index changes gradually as a function of position within the material. GRIN materials have several advantages over traditional materials with uniform refractive indices. Traditional lenses have spherical aberration because electromagnetic rays passing through their edges are refracted differently than electromagnetic rays passing through their centers. Spherical aberration can be lessened with graded-index materials by gradually changing the refractive index of the lens from its center to its edges. In comparison to conventional materials, GRIN materials can be created to have a refractive index profile that is more uniform, resulting in better quality. GRIN materials can be created with a refractive index that is similar to the medium it is in, reducing reflection at the material interfaces. As they can bend electromagnetic materials more effectively than conventional materials, graded-index materials can enable more compact designs for electromagnetic devices by eliminating the need for multiple lenses. As a result, there is an urgent need to develop a GRIN dielectric-based flat lens that is isotropic, compact, wideband and easy to manufacture. 

A compatible WB antenna is also necessary for the proposed lens design to operate over a wide impedance bandwidth. In the literature, several techniques have been reported to increase the bandwidth of printed antennas at the millimeter-wave (mm-wave) band. However, such techniques added complexity to the antenna structure. For example, the technique used in [11] relies on the excitation of two intrinsic modes, which requires the use of an additional dielectric layer to excite the higher-order modes. The resulting −10 dB impedance bandwidth of 22.5% was obtained in the range of 24.1–30.2 GHz. However, a good impedance bandwidth was achieved at the cost of a complex antenna profile. Similarly, in [12], an inset-fed patch antenna with an additional parasitic patch layer was presented to be operated from 25.55 to 29.75 GHz with a relatively high gain value of 8.2 dBi at 28 GHz. The additional complexity was the parasitic patch layer that increases the profile height by 300%, from 0.254 mm to 1.004 mm. 

In [13], multi-layer parasitic patches were utilized to enhance the bandwidth. However, due to the use of multiple PCB layers, the good impedance bandwidth was achieved at the expense of increasing the antenna’s size. In [14], additional zero-modes were created by connecting the patch together, resulting in an operating bandwidth of 24.5–28 GHz, with a lower gain of 6–7 dBi. In another relevant design, as reported in [15], an impedance bandwidth of 26.65–29.14 GHz was obtained using a dual-slot-fed patch antenna based on a substrate-integrated waveguide. The gain value reported was 6.4 dBi at 28 GHz.

In this paper, a widening-band antenna with a GRIN 2D dielectric lens is designed for 5G applications. This lens is made by drilling holes in a flat dielectric in an uneven pattern to obtain an effective refractive index that is similar to that of the Luneburg lens. A compatible WB antenna is designed to work with the designed lens over a wide impedance bandwidth. The antenna dimensions and the thickness and overall dimensions of the lens are optimized with the goal of creating a compact lens antenna with optimal performance (impedance matching bandwidth, gain, 3 dB beamwidth, and sidelobe level).

## 2. Patch Antenna Design

In this work, a microstrip patch antenna is designed to cover a wideband (WB) frequency span to cover the 5G mm-wave standards. For 5G mm-wave applications, the lens must work over a wide frequency range. This means that it needs a WB feeding mechanism. Since the proposed lens design is WB by nature, the feeding mechanism must also be WB-compatible. The optimized integrated lens and patch antenna setup works well over the targeted band, with high gain values and stable performance over the whole band of operation.

**Figure 1:**
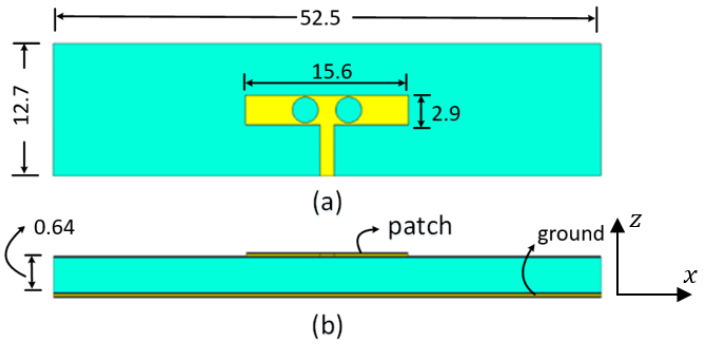
WB patch antenna: (**a**) top view; (**b**) front view—all dimensions are in millimeters (mm).

As shown in Figure 1, the proposed antenna design was a square patch with two circular slots. The antenna is designed using a two-layer substrate board of Rogers RT5880 with relative permittivity ε_r_ = 2.2, and loss tangent tanδ = 0.0009. The PCB board used has dimensions of 12.7 × 52.5 mm^2^ and a thickness of 0.64 mm. The rectangular patch size is 15.6 × 2.9 mm^2^ on the top layer. Two circular slots are etched out of 2.5 mm. The bottom layer of the PCB board serves as a reference ground plane for the rectangular patch. The proposed patch antenna is fed via a microstrip line of 1.4 mm width. Figure 1 depicts the dimensions of various patch antennas.

To achieve a wide range of impedance, the patch was optimized without any slots by choosing the right patch size. Parametric sweeps are performed to analyze the effect of the width and length of the patch antenna. Both slots are identical and positioned symmetrically around the antenna’s center and they are far away from each other by 4.2 mm. To achieve wideband impedance matching and minimize return loss, the input impedance of the antenna is set to 50 ohms by optimizing the width and length of the feeding microstrip line. As per our findings, the slots created an extra resonant mode at the lower frequency end, which helped in widening the operating bandwidth. The final optimized dimensions resulted in an increased effective bandwidth of the patch in the range of 26–30.5 GHz.

**Figure 2:**
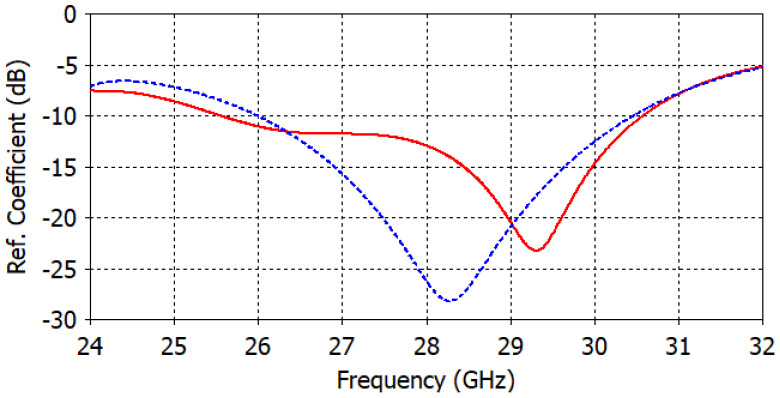
The reflection coefficient of the proposed microstrip patch antenna using FIT (solid line) and FEM (dotted line) solvers.

**Figure 3:**
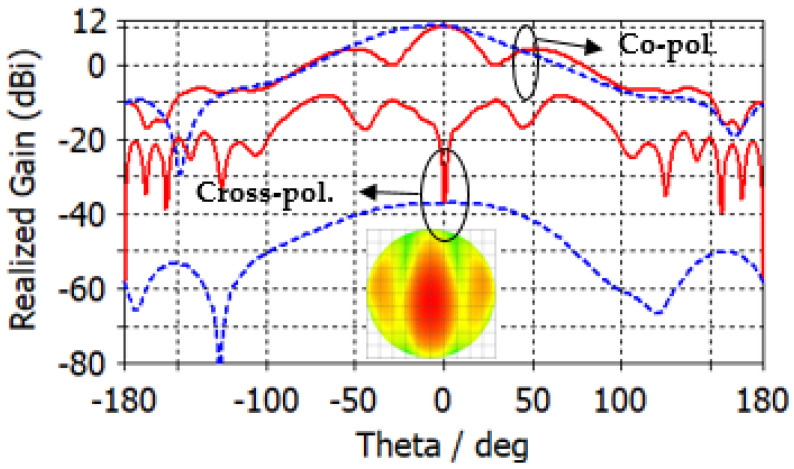
Co-polarized and cross-polarized realized gain of the proposed microstrip patch antenna at 28 GHz for $\phi =0^{{}^{\circ}}$ (solid line) and $\phi =90^{{}^{\circ}}$ (dashed line).

**Figure 4:**
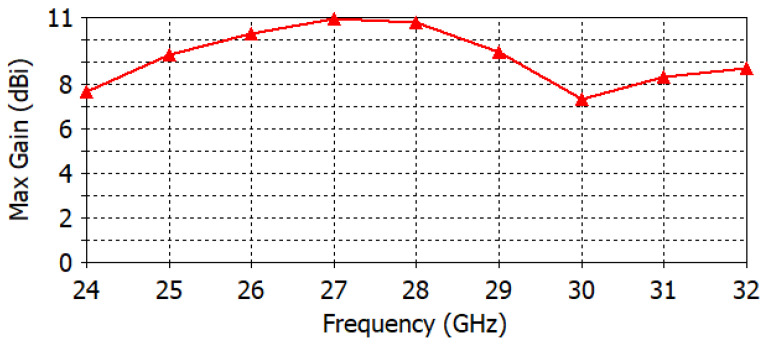
The maximum gain over frequency.

The antenna port is excited by a Gaussian pulse, polarized along the *z*-axis and propagating along the +*y*-axis, as shown in Figure 1b. The numerical solution is performed for open boundaries of the electromagnetic problem (far-field problem) using the finite integration technique (FIT) solver in CST. The simulation results are validated using two different full-wave solvers, which are the finite integration technique (FIT) and the finite element method (FEM) solvers in CST. Figure 2 shows the reflection coefficient curve of the proposed microstrip patch antenna using both FIT (solid line) and FEM (dotted line) solvers. From the given figure, it is evident that the antenna is operating over a wideband with a −10 dB impedance bandwidth of 26–30.5 GHz for both solvers. Figure 3 depicts the co-polarized and cross-polarized realized gain of the proposed patch antenna at 28 GHz for the following two different azimuthal angles: $\phi =0^{{}^{\circ}}$ (solid line) and $\phi =90^{{}^{\circ}}$ (dashed line). The results indicate that the co-polarized gain is generally higher than the cross-polarized gain, suggesting that the co-polarized radiation primarily determines the radiation direction of the antenna. The orthographic radiation pattern at 28 GHz is also shown in Figure 3, which shows an asymmetric focus of radiation in elevation and azimuthal planes. Figure 4 illustrates the maximum realized gain with frequency. The results reveal that the antenna achieves a maximum gain of 11 dBi at a frequency of 27.5 GHz. This value is a crucial parameter in assessing the antenna’s performance, as it indicates the maximum achievable gain for the given operating frequency. 

## 3. GRIN Dielectric Lens Design

A subwavelength unit cell is used to obtain the effective refractive index, which is the first step in designing a GRIN dielectric lens. The suggested unit cell dimensions in both the x and y directions are 3 mm (0.28 λ). The thickness of the unit cell is 1.57 mm (0.1493 λ). The reason for the selection of the value of 1.57 is the commercial availability of Rogers TMM 6 (ε_r_ = 6.3). For normal incident plane waves on the slab, the relationships between the complex refractive index ($n_{eff}$) and the scattering parameters [16] are given by

(1) $$n_{eff}=\frac{-i}{\kappa d}\;ln\left(\frac{S_{11}}{1-S_{21}\left(\frac{z_{eff}-1}{z_{eff}+1}\right)}\right)$$

where $S_{11}$ is the reflection coefficient, $S_{21}$ is the transmission coefficient, d is the lens slab thickness, *k* is the free-space wavenumber and $z_{eff}$ is the complex effective wave impedance, which is given by the following equation:

(2) $$z_{eff}=\pm \sqrt{\frac{{\left(1+S_{11}\right)}^{2}-S_{21}{}^{2}}{{\left(1-S_{11}\right)}^{2}-S_{21}{}^{2}}}$$

For passive materials, the real value of $z_{eff}$ and the imaginary part of the refractive index, $n_{eff}$, must be greater than or equal to zero.

**Figure 5:**
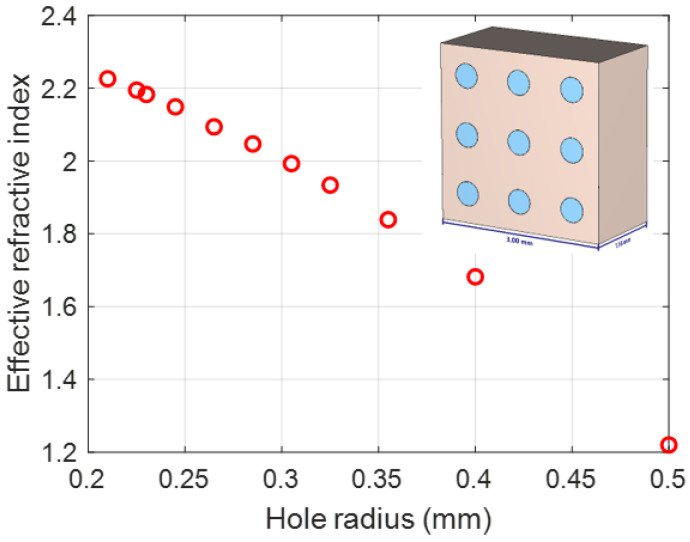
The graded effective refractive index versus hole radius.

**Figure 6:**
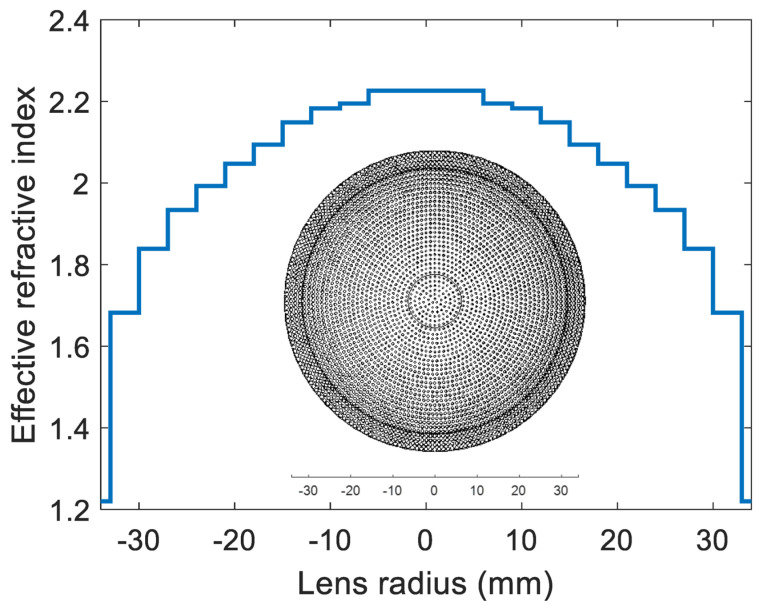
The graded effective refractive index versus lens radius.

Figure 5 shows the calculated effective refractive index for different hole radii based on Equation (1). To design a compact graded effective refractive index lens, optimization is performed to obtain the smallest possible design, and also to obtain lower applicable values of the effective refractive index to avoid the considerable reflections due to the impedance mismatch. The refractive index profile of a proposed lens is similar to that of the Luneburg lens [7], except for the minimum (at the end of the lens) and maximum (at the center of the lens) values of the effective refractive index. In the Luneburg lens, the refractive index profile changes from 1 to 2. In the proposed lens, the minimum and maximum values are approximately 1.2 and 2.25, respectively. The maximum gain and minimum sidelobe levels are also criteria for the selection of the minimum thickness of a flat lens (8 mm). The final diameter of the lens design is reduced to 34 mm (3.1733 λ), with a focal length of 5.97 λ and thickness of 5 × 1.6 mm (0.746 λ). The designed dielectric flat lens consists of a set of eleven concentric rings of different refractive indices, to produce the desired phase delays required to obtain the desired gain, sidelobe level, and 3 dB beamwidth. Figure 6 shows the graded effective refractive index versus the lens radius. This lens is thinner than the conventional existing shaped lenses.

**Figure 7:**
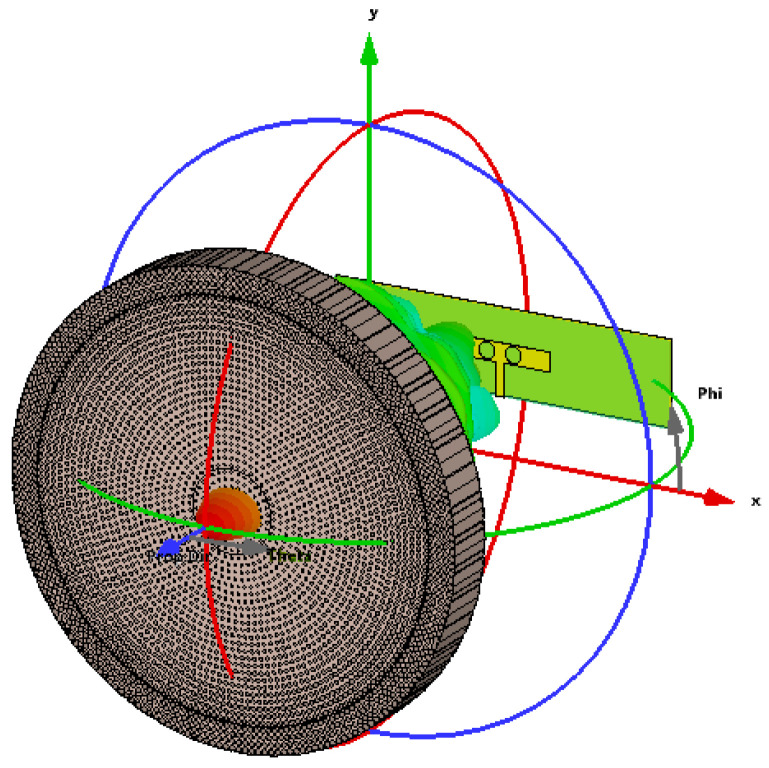
The designed GRIN dielectric lens antenna.

**Figure 8:**
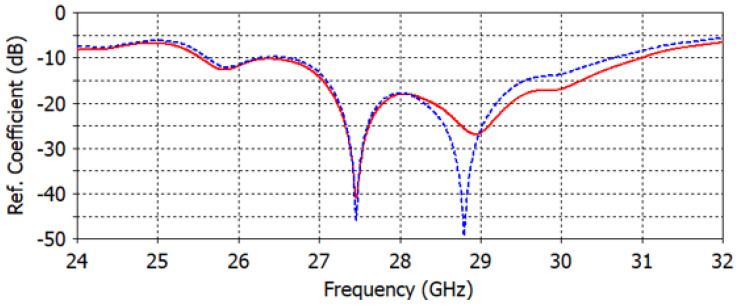
The reflection coefficient of the proposed antenna lens using FIT (solid line) and FEM (dotted line) solvers.

When the lens is added to the microstrip patch antenna as shown in Figure 7, the proposed dielectric lens allows the antenna to work better. The return loss (Figure 8) is still lower than −10 dB for the selected frequency around 28 GHz for both FIT (solid line) and FEM (dotted line) solvers. The interpretation of the small variation in the reflection coefficient of around 28 GHz involves choosing the smallest possible values of the graded refractive index (1.2 to 2.2), which ensures that the designed lens is approximately matched to the air layer.

**Figure 9:**
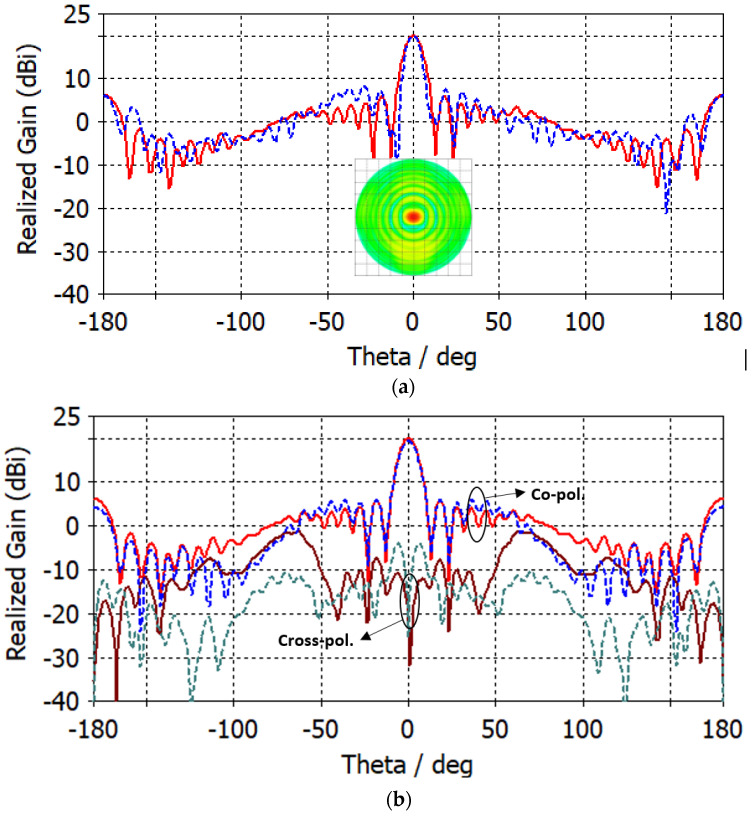
(**a**) Realized gain of the proposed patch antenna with lens for $\phi =0^{{}^{\circ}}$ (solid line) and $\phi =90^{{}^{\circ}}$ (dashed line); (**b**) co-polarized and cross-polarized realized gain of the proposed patch antenna with lens for $\phi =0^{{}^{\circ}}$ using FIT (solid line) and FEM (dotted line) solvers.

Figure 9a shows the realized gain of the patch antenna with the proposed dielectric lens for $\phi =0^{{}^{\circ}}$ (solid line) and $\phi =90^{{}^{\circ}}$ (dashed line). The maximum gain at 28 GHz after adding a flat dielectric lens increased from 10.8 dBi (see Figure 3) to 20 dBi for both $\phi =0^{{}^{\circ}}$ (solid line) and $\phi =90^{{}^{\circ}}$ (dashed line). The sidelobe level is also −14 dB and −12 dB for $\phi =0^{{}^{\circ}}$ (solid line) and $\phi =90^{{}^{\circ}}$ (dashed line), respectively. The angular width is $9.6^{{}^{\circ}}\;$ and $7.8^{{}^{\circ}}$ for $\phi =0^{{}^{\circ}}$ (solid line) and $\phi =90^{{}^{\circ}}$ (dashed line), respectively. The 2D orthographic projection of the radiation pattern is shown in the same figure. The orthographic projection of the radiation pattern of a lens antenna (appears in the same figure) is projected onto a plane that is orthogonal to the antenna’s main radiation axis. The resulting image shows the relative strength and directionality of the radiation pattern as viewed from a particular angle ($\phi =0^{{}^{\circ}}$). Figure 9b shows the co-polarized and cross-polarized realized gain of the proposed patch antenna with lens for $\phi =0^{{}^{\circ}}$ at 28 GHz, using both FIT (solid line) and FEM (dotted line) solvers. By comparing the simulation results based on the FIT solver and those of the FEM solver, we can observe that the simulated results are in good agreement. It is also observed that the co-polarized gain of the antenna is generally stronger than the cross-polarized gain. Therefore, the co-polarized radiation plays a dominant role in the radiation direction of the lens antenna. 

**Figure 10:**
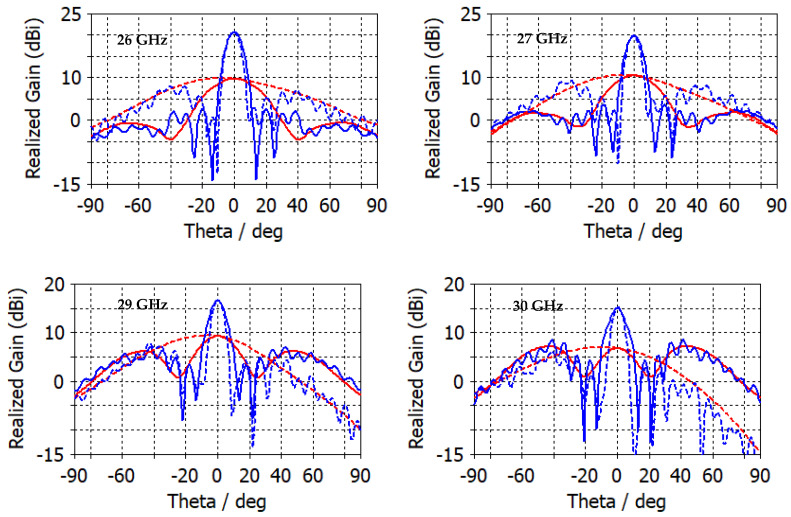
The 2D realized gain of the proposed patch antenna with (blue color) and without (red color) a lens at different frequencies, and for $\phi =0^{{}^{\circ}}$ (solid line) and $\phi =90^{{}^{\circ}}$ (dashed line).

**Figure 11:**
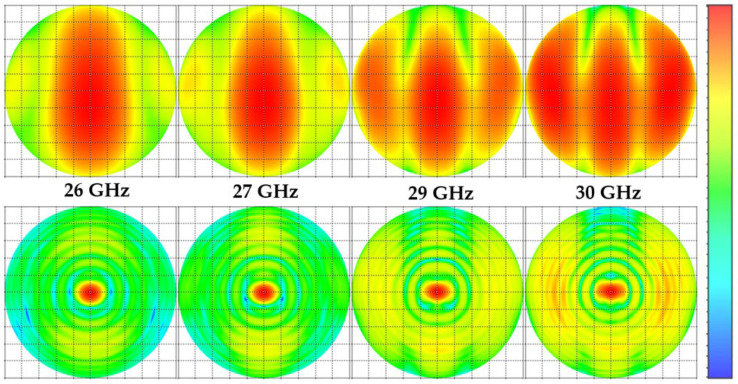
Orthographic radiation patterns with (down) and without (up) a lens at different frequencies.

Figure 10 shows the 2D radiation pattern of the realized gain for frequencies, 26 GHz, 27 GHz, 29 GHz, and 30 GHz. The maximum gain after adding a flat dielectric lens increased from 9.7 dBi to 20.7 dBi at 26 GHz, from 10.47 dBi to 19.8 dBi at 27 GHz, from 9.7 dBi to 20.7 dBi at 29 GHz and from 9.25 dBi to 16.6 dBi at 30 GHz. The angular width for $\phi =0^{{}^{\circ}}$ is $9.8^{{}^{\circ}}\;$ for 26 GHz, $9.6^{{}^{\circ}}$ for 27 GHz, $9.8^{{}^{\circ}}$ for 29 GHz and $10.3^{{}^{\circ}}$ for 30 GHz, whereas for $\phi =0^{{}^{\circ}}$, it is $8.5^{{}^{\circ}}\;$ for 26 GHz, $7.9^{{}^{\circ}}$ for 27 GHz, $7.5^{{}^{\circ}}$ for 29 GHz and $7.4^{{}^{\circ}}$ for 30 GHz. The orthographic radiation pattern with and without a lens at different frequencies (see Figure 11) shows how much the flat lens focuses the radiation pattern in a small region at the center of the view. The specific shape and characteristics of the orthographic projection of a lens antenna’s radiation pattern depend on the design of the lens and the characteristics of the antenna. The radiation pattern of a lens antenna is characterized by high gain, directivity, and a narrow beamwidth in the direction of the lens axis compared to that of the antenna alone. The specific details of the radiation pattern vary depending on the specific design parameters of the lens antenna, such as the refractive index profile of the lens and the geometry of the antenna structure.

**Figure 12:**
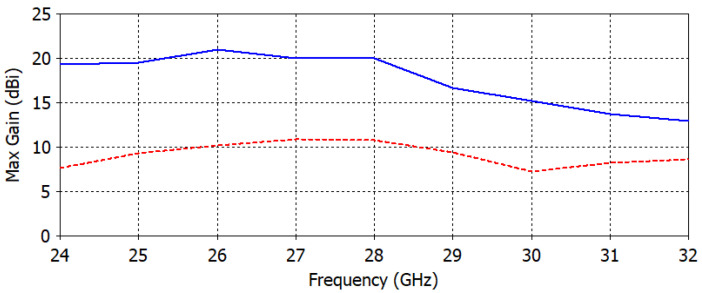
Maximum gain of the proposed patch antenna with a lens (dashed line) and without (solid line).

Figure 12 compares the maximum gain of an antenna with and without a lens over a range of frequencies. The antenna with a lens is designed to improve the directivity and gain of the antenna by focusing the radiation pattern in a particular direction. The results show that the antenna with the lens has a significantly higher maximum gain compared to the antenna without the lens. The comparison highlights the effectiveness of using a GRIN dielectric-based flat lens to improve the performance of an antenna for a specific frequency range. 

A comparison of several key metrics of the proposed dielectric slab-based lens antenna with previously published results is shown in Table 1, showing that our proposed lens antenna is more favorable in terms of gain, quality factor, thickness, largest dimension, and the type of lens and antenna with those produced using more expensive fabrication methods and high-cost and dense dielectric materials and metamaterials. The proposed lens antenna has a lower Q factor compared to some other antennas, which results in a wider bandwidth, except for the design in [17], which utilized a complex printed antipodal log-periodic dipole array antenna. Furthermore, the proposed lens has the lowest thickness, which offers several advantages in antenna design, such as a reduced weight and size, and simplification of the design and manufacturing process, making it easier and less expensive to produce.

## 4. Conclusions

In this work, a GRIN dielectric lens has been proposed to meet the high-gain requirements of 5G applications. The proposed design is based on the effective refractive index of a hole-type unit cell. An WB antenna is also designed to be compatible with the lens operation over a wide impedance bandwidth. The proposed integrated patch along with the lens is tested for its performance using a numerical full-wave simulator. The patch-lens system demonstrated a gain of 20 dBi, HPBW of $9.6^{{}^{\circ}}\;$ and $7.8^{{}^{\circ}}$ for $\phi =0^{{}^{\circ}}$ and $\phi =90^{{}^{\circ}}$, respectively, a low sidelobe level of around −14 dB and −12 dB for $\phi =0^{{}^{\circ}}$ and $\phi =90^{{}^{\circ}}$, respectively, for the antenna placed in the center, and a focal point of 64 mm. The simulation results were validated using the finite element method solver, and the proposed system’s performance was found to be well-matched with the high-gain and wideband requirements of 5G antenna systems.

The proposed lens antenna system provides several benefits, such as improved performance, compactness, and low-profile design. It also has the potential to be extended to other frequency bands and applications, making it a versatile and cost-effective solution for wireless communication systems.

## Figures and Tables

**Table 1:** Comparison of the proposed wideband GRIN dielectric lens antenna with previously published results.

	Maximum Gain	The Q Factor	Resonance Frequency	Lens Thickness	Largest Dimension	Designed Lens	Designed Antennas
This work	20 dBi	6.2	28 GHz	0.746 λ	3.1733 λ	GRID dielectric lens	Microstrip patch antenna
[17]	11.9 dBi	2.83	17 GHz	2.71 λ	2.71 λ	3D-printed comb-mushroom-like dielectric lens	Printed antipodal log-periodic dipole array antenna
[18]	10.12 dBi	34.11	5.8 GHz	1.4 λ	1.4 λ	3D-printed dielectric lens	A circular microstrip patch antenna
[19]	12.1dBi 15.6 dBi	18.6 9.33	28 GHz 28 GHz	1.5 λ 3 λ	1.5 λ 3 λ	Semi-spherical dielectric lens	Microstrip patch antenna (MPA) array and substrate integrated waveguide slot antenna array
[20]	16.4 dBi	20	10 GHz	1.4 λ	1.4 λ	Metasurface layered lens	Patch antenna
[21]	19.25 dBi	12.63	60 GHz	2.3 λ	2.3 λ	Extended hemispherical-shaped, homogenous dielectric lens	Rectangular microstrip antenna
[22]	17 dBi	8.5	77 GHz	1.4 λ	5 λ	GRID dielectric lens	Conical horn antenna

## Data Availability

Not applicable.
